# A Quality Improvement Project to Improve Documentation and Awareness of Limitations of Life-Sustaining Therapies

**DOI:** 10.1097/pq9.0000000000000304

**Published:** 2020-05-28

**Authors:** Amy H. Jones, Julia A. Heneghan, Bonnie Brooks, Mia Maamari, Ali Ahmad, Tessie W. October, Christiane Corriveau

**Affiliations:** From the * Department of Critical Care, Children’s National Hospital, Washington, D.C.; † Department of Pediatrics, The George Washington University School of Medicine and Health Sciences, Washington, D.C.

## Abstract

**Purpose:**

Poor documentation and understanding of the limitations of life-sustaining therapies upon admission to the pediatric intensive care unit (PICU) can result in moral distress for both providers and families. Limitations of life-sustaining treatments are often not documented and/or understood by members of the health care team.

**Methods:**

We performed a quality improvement initiative to improve the care teams’ understanding and paper documentation of the limitations of life-sustaining therapies in the PICU of a quaternary children’s hospital from January 2018 to March 2019. We implemented a series of plan-do-study-act cycles, including initiation of an updated rounding tool that included limitations of interventions, in-person and electronic information sessions, and implementation of a visual bedside tool to remind providers when limitations were present. Pre- and postintervention surveys were administered.

**Results:**

Nursing paper documentation of limitations of life-sustaining therapies increased sequentially from 0% to 88% during plan-do-study-act cycles. Creating a specific area to document limitations on the nursing sheet resulted in the most significant increase in documentation (36.6 points). Nurses reported that they “always” document limitations, which increased from 10% to 38%. The percentage of nurses who understood patients’ intervention limitations increased from 28% to 33%.

**Conclusions:**

Limitations of life-sustaining therapies in the PICU are nuanced and involve multiple stakeholders. Nursing education and designation of a section of intervention limitations in nursing daily goal paper documentation can increase comfort with therapeutic limitations in the PICU. Future studies should explore impacts on patient care and serve as a framework for the ultimate goal of improving documentation of care limitations and code status in the electronic medical record.

## Abstract 2

Supplemental Digital Content is available in the text.

## INTRODUCTION

Decision-making at the end of life for infants and children is among the most difficult experiences families will encounter and among the most important and profound professional responsibilities for the clinicians who care for them.^1,2^ As pediatricians, our medical care is guided by the child’s best interest, which in most circumstances leads us to make every effort to sustain life. However, there are situations in which the burdens of interventions to sustain life outweigh the benefits to the child.

In a policy statement from 2017, the American Academy of Pediatrics recommended favoring interventions that are likely to provide greater benefit than burden for the child and discouraging the initiation or continuation of interventions that are likely to lead to a greater burden than benefit.^3^ The role of the pediatric intensive care unit (PICU) team is to partner with families to ensure families are aware of a child’s prognosis and the therapeutic options that may be beneficial or futile based on the clinical scenario. Multiple studies have demonstrated that families who make joint decisions with a child’s care team regarding end-of-life decisions have improved satisfaction with their child’s care and bereavement outcomes.^4–7^

Despite these benefits, the discussion and documentation of family and patient decision-making regarding the end of life are inconsistently achieved in the pediatric population. In a prospective pediatric cohort study examining deaths across 5 teaching hospitals in the United States, Burns et al^8^ observed that 70% (133/192) of pediatric patients died following withdrawal or withholding of life-sustaining therapy. Interestingly, of those who died as the result of withholding or withdrawing support, only 64% had a formal do not resuscitate (DNR) order in place. An Australian single-center retrospective chart review of pediatric patients admitted in 2011 with life-limiting conditions found that only 10% (4/40) of patients with limitations of life-sustaining therapies in place had limitations easily located within the medical record.^9^

These studies identify deficiencies in the designation and documentation of the limitations of interventions, which may have implications in the medical team’s understanding of the goals of care. Although the DNR order has existed in a variety of forms for several decades, the DNR order continues to be a source of anxiety and confusion for both families and care teams as medical and surgical technologies advance.^10,11^ This anxiety and confusion may limit understanding of the details of a specific family’s wishes for the care of their child, resulting in repetitive conversations with families regarding goals and priorities, or provision of therapies not consistent with family wishes.

**Fig. 1.**
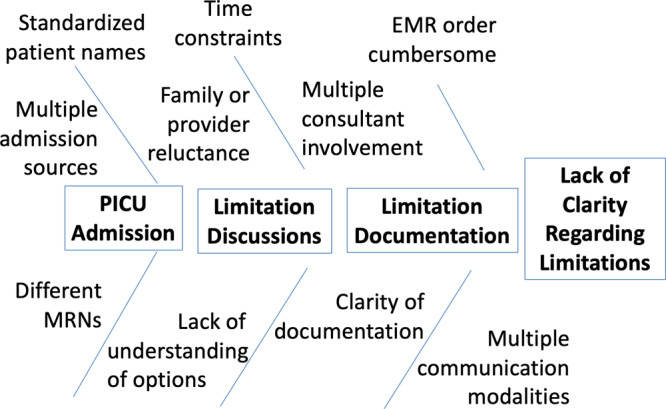
Ishikawa cause and effect diagram.

At our institution, there was a lack of clarity regarding code limitations and concern for inadequate documentation of code status. This deficiency prompted further exploration and, ultimately, led to this quality improvement initiative. We created an Ishikawa *cause and effect* diagram to identify barriers to code status documentation at the time of PICU admission (Fig. 1). One of the major barriers identified was the cumbersome and time-consuming process of locating limitations of life-sustaining interventions within the electronic medical record (EMR). To streamline the efficiency in locating limitations as well as in an attempt to facilitate conversations regarding limitations on rounds, we focused on modification of the “Daily Goal Sheet,” a paper rounding tool, rather than the EMR, as one component of the intervention. The global aim was to improve the quality of supportive care provided for patients and families by improving code status documentation among bedside staff. The secondary aim was to improve understanding of limitations of life-sustaining therapies and their importance by the bedside nurse.

## METHODS

**Fig. 2.**
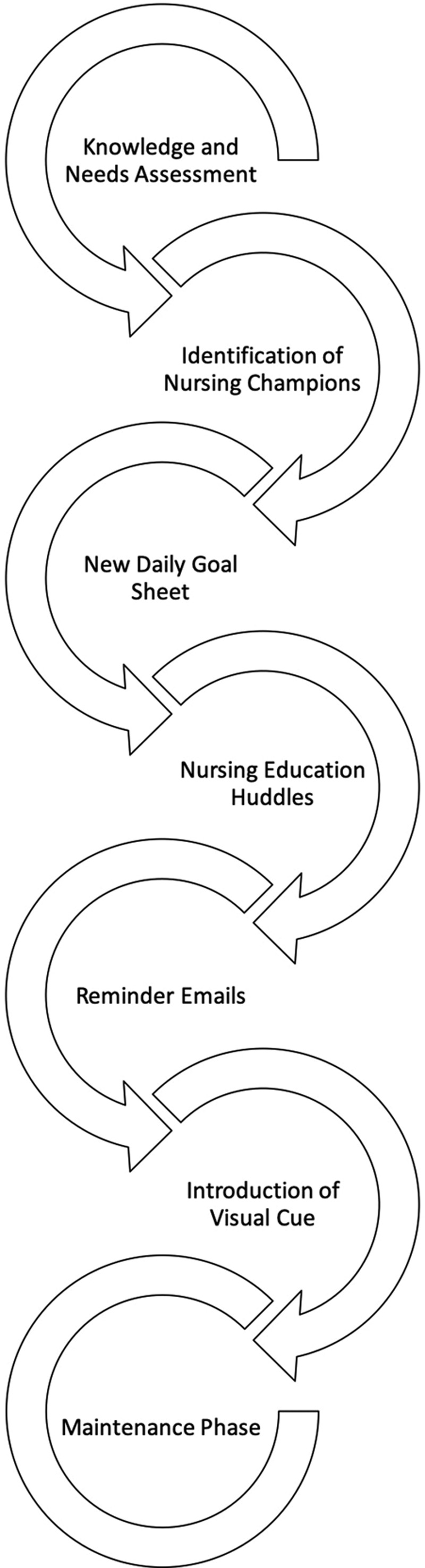
PDSA cycle graphic.

This project was a quality improvement initiative where 5 “plan-do-study-act” (PDSA) cycles were completed based on a SMART (Specific, Measureable, Achievable, Realistic and Timely) aim. A SMART aim is used in quality improvement initiatives to ensure that the intervention is “specific, measurable, attainable, relevant, and time-bound.”^12^ The SMART aim for this project was to increase code status documentation and understanding from 0% to 80% in the PICU over 14 months from January2018 to March 2019 (Fig. 2).

Before starting the PDSA cycles, we distributed a survey to pediatric ICU nurses who provide the primary bedside care to patients in our unit (Survey, Supplemental Digital Content 1, available at http://links.lww.com/PQ9/A186). This survey was developed with the aid of nursing champions to assess baseline knowledge of limitations of life-sustaining therapies and functioned as a needs assessment. We established face validity with critical care and palliative care physician experts. The results of the baseline knowledge and needs assessment were used to develop educational materials related to end-of-life care in PICU patient. The PDSA cycles included (1) identifying nursing champions, (2) adding limitations of life-sustaining therapies section to a structured daily rounding tool, (3) nursing education regarding the nuances of limitations of life-sustaining therapies discussions and documentation, (4) reminder emails with educational materials, and (5) creation of a visual cue.

**Fig. 3.**
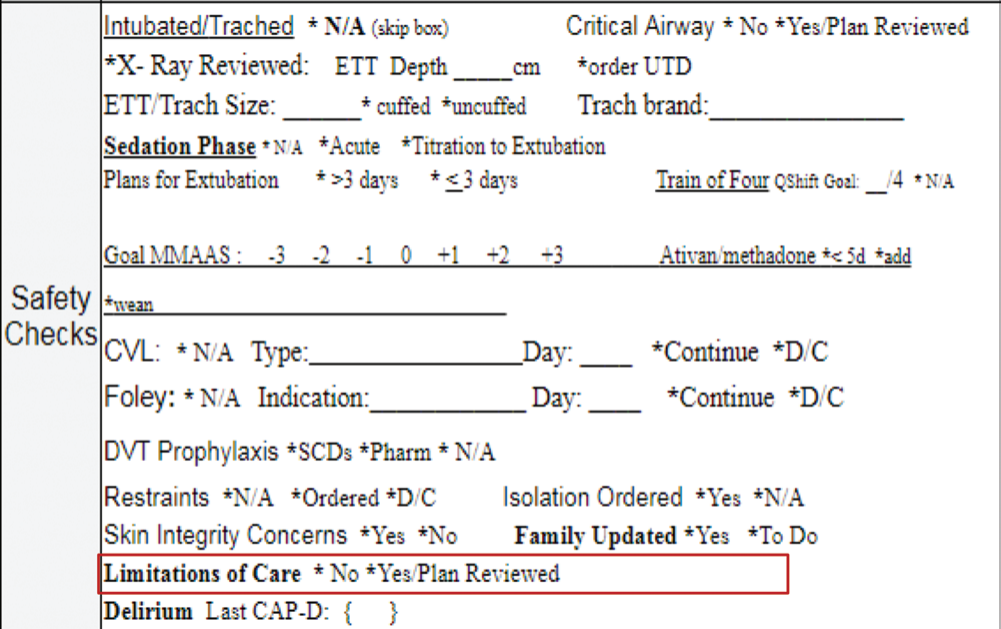
Limitations of care portion of Daily Goal Sheet. CAP-D, Cornell Assessment of Pediatric Delirium; CVL, central venous line; D/C, discontinue; DVT, deep venous thromboembolism; MMAAS, Modified Motor Activity Assessment Scale; N/A, not applicable; SCD, sequential compression device; UTD, up to date.

We initially identified 3 experienced nursing champions to support our efforts and promote continuity of nursing education. The nurse champion provided feedback to the physician team regarding the educational interventions and implementation of the Daily Goal Sheet. They also provided feedback on how the visual tool (Visual Tool, Supplemental Digital Content 2, available at http://links.lww.com/PQ9/A187) was perceived by the bedside staff and helped provide reminders as to the importance of using this tool. In our unit, the Daily Goal Sheet is a structured tool completed twice a day by the PICU nurses for each patient to summarize the plan of care for the shift. There was no specific area to document the limitations of life-sustaining therapies on this tool before the quality improvement initiative. A portion of the tool is dedicated to a series of “safety checks,” such as the presence of a Foley catheter and central lines, the provision of deep venous thromboembolism prophylaxis, and other aspects of routine ICU care. A yes/no indicator for “limitations of care present” was added to this portion of the tool (Fig. 3). We anticipated that the statement of “limitations of care” on rounds would distress the family and/or the nurses and assessed this as a balancing measure.

Educational sessions, focused on supportive and family-centered care, were held at morning and evening nursing huddles. These sessions introduced the expectations for documentation, as well as discussed the importance and characteristics of limitations of interventions. Following the implementation of the new Daily Goal Sheet format, reminder emails were sent to physicians and nursing providers to encourage its use. Ultimately, a visual cue was introduced at the bedside to remind providers when a patient had limitations of life-sustaining therapies in place. This cue, a picture of a caterpillar, was designed as a laminated card that was to be placed outside of individual patient rooms. The maintenance phase included additional reminder emails and a postintervention survey to elicit a change in knowledge as well as overall project feedback. Finally, members of the Quality Improvement (QI) team observed morning rounds for 2 separate 1-week periods (February and March 2019). They recorded the presence of documentation in the limitations of life-sustaining therapies section on the Daily Goal Sheet and whether or not limitations were stated on rounds.

Our primary aim was to increase the percentage of Daily Goal Sheets with the “limitations of care” section documented from 0% to 80%. A total of 924 patient-days of documentation were reviewed on a convenience basis during selected months for patients admitted over 14 months from January 2018 to March 2019. There were no exclusion criteria. The primary outcome measure used to assess this outcome was documentation completion frequency of “limitations of care” on Daily Goal Sheets for all pediatric ICU admissions.

Our secondary outcome measure was the improvement in PICU nursing staff understanding of the limitations of life-sustaining therapies and patient–family centered care. The process measure used to assess this outcome was self-reported nursing staff understanding and comfort regarding the limitations of interventions, as well as descriptions of nursing workflow regarding the communication of limitations of interventions. We collected survey data via REDCap (REDCap, Nashville, Tenn.). Data were analyzed using Microsoft Excel (Microsoft Corporation, Redmond, Wash.). This study was reviewed and approved by the Institutional Review Board at the Children’s National Hospital.

## RESULTS

### Baseline Nursing Surveys

A total of 41 nurses out of 92 (44.6%) completed the preintervention survey. The majority of respondents strongly agreed (24.4%) or agreed (61.0%) with the statement, “I fully understand my patients’ limitations of care.” Most participants (56.1%) reported never using signage or visual cues to remind the team when limitations of life-sustaining therapies were present, while about one-third (34.1%) of participants reporting sometimes using these cues.

More than half (56.0%) of nurses reported “never” documenting a patient’s limitations of intervention status. When specifically asked how information related to the limitations of intervention was relayed during nursing handoff, 10% reported the Daily Goal Sheet was used as a framework to ensure complete handoff. The vast majority (85.4%) of participants were in favor of a universal visual tool to indicate when a patient has limitations of life-sustaining therapies in place. In the open-ended portion of the survey, participants reported concerns over how limitations of life-sustaining therapies were relayed shift to shift and the accuracy of that information. Nurses’ open-ended suggestions for improvements included “we should add code status to daily rounds sheet!” and “some sort of sign or visual cue would be great (even hospital-wide!) and would help in cases where the patient’s bedside nurse or parent is not present to remind people of Allow Natural Death/DNR status.”

### Postintervention Nursing Surveys

A total of 35 participants out of 92 (38%) completed the postintervention survey. The majority of participants reported they either strongly agreed (28.6%) or agreed (48.6%) with the statement of understanding a patient’s limitations of interventions. Postintervention, 40.0% of participants reported always documenting limitations of life-sustaining therapies on the Daily Goal Sheet and 45.7% reported sometimes documenting these limitations. The vast majority of participants (91.4%) were aware of the visual tool created to remind bedside providers when limitations of life-sustaining therapies were in place. In open-ended response questions, nurses requested more granularity regarding specific patient-level interventions and the robust involvement of the palliative care team.

### Documentation of Limitations of Care

**Fig. 4.**
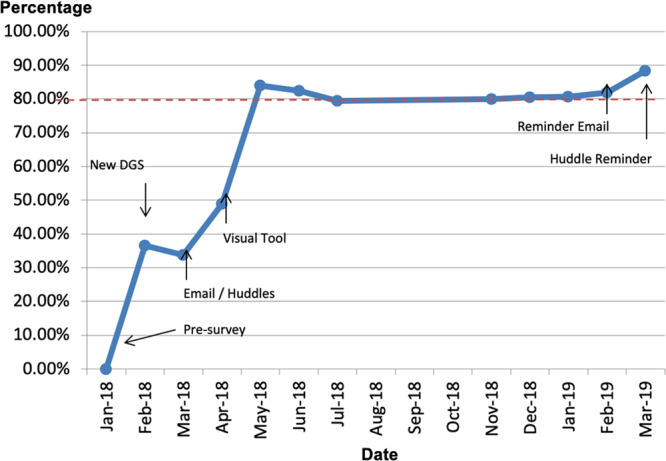
Run chart.

Written documentation of limitations of life-sustaining therapies on the Daily Goal Sheet increased sequentially from 0% to 88% throughout the PDSA cycles (Fig. 4). The creation of a specific area to document the limitations of life-sustaining therapies on the nursing sheet resulted in the largest increase in documentation (36.6 points). Documentation of limitations of life-sustaining therapies continued at a steady rate throughout the maintenance phase of the project, remaining above the goal of 80% documentation compliance for 10 months, indicative of a centerline shift.

During the maintenance phase, adherence to the documentation of limitations on the Daily Goal Sheet was directly observed by a critical care fellow who was already on rounds for that day. A total of 160 patient-days over two 1-week periods were observed. The bedside nurse read the “limitations of care” section without prompting 94% (150/160) of the time. In the remaining 10 observations, the Daily Goal Sheet was not read, and thus safety checks, including limitations of life-sustaining therapies, were not reported spontaneously. When a QI team member prompted the bedside nurse to report these safety checks, the limitations of care section was subsequently stated 60% (6/10) of the time. During these observation periods, a single patient had limitations of life-sustaining therapies in place. For this patient, the Daily Goal Sheet was completed, with limitations of life-sustaining therapies documented 93% (13/14) of the time and stated aloud on rounds 100% (14/14) of the time.

## DISCUSSION

We demonstrated a significant improvement in the written documentation of limitations of life-sustaining therapies during this initiative, largely due to the implementation of a structured nursing tool. This result justified a centerline shift and trend toward significance during this 14-month study period. We were also able to increase the number of nurses who “strongly agreed” with their understanding when a patient had limitations of life-sustaining therapies in place. We also created a visual tool and successfully implemented it into the PICU environment when a patient had limitations of life-sustaining therapies present. Using the Daily Goal Sheet to record the limitations of life-sustaining therapies allowed for incorporation into the daily workflow and provided a peer-check opportunity between nursing and physician staff members on rounds. Aligning with our nursing champions for this initiative enhanced nursing education.

This project impacted the people and systems through the use of a visual prompt and section on the Daily Goal Sheet, resulting in the integration of the limitations of intervention awareness into our daily workflow. This trigger served to prompt and facilitate discussion between the family and medical team, particularly if there was a lack of clarity or a need to change a patient’s limitations. Interestingly, although the percentages of nurses who strongly agreed with understanding their patient’s code status increased (24.4%–28.6%), the percentage who agreed decreased (61.0%–48.6%). The explanation for this decrease is likely multifactorial. One possible explanation is that new and/or different nurses completed the postintervention survey who did not yet receive the educational sessions and emails outlining the quality improvement project. Additionally, the QI team’s emphasis on the importance of aligning with family goals and values more globally rather than providing nursing staff–specific and concrete goals of care (ie, titrate epinephrine to a maximum of 0.2 μg/kg/min) may have led to more confusion among newer nurses.

The visual cue also helped subjectively provide situational awareness for any care providers visiting the bedside by reducing steps needed to verify the presence of limitations of interventions, particularly in an emergent situation where comfort and resuscitation would take divergent paths at the bedside. We had anticipated that the statement of “limitations of care” on rounds would distress the family and/or the nurses and could serve as a balancing measure. However, anecdotally, we have not encountered this issue. The educational sessions that served to introduce the new Daily Goal Sheet and explain the reasoning for its need also helped mitigate potential concerns about mentioning limitations of care.

### Limitations

Although we achieved a consistent trend of improvement in documenting the limitations of life-sustaining therapies in our institution, this quality improvement project was only implemented in a single unit at a single center. It may not be replicable in other units or centers. Although the Daily Goal Sheet is used frequently in our unit, not all nurses use this method of documentation, and it is not a part of the formal medical record. We were unable to examine other, more informal ways of documenting the limitations of life-sustaining therapies that may have been used by bedside providers. Additionally, since we collected data daily (not patient basis), patients with long lengths of stay are likely overrepresented in the sample. Inherent to survey data, our results may have selection bias, reflecting the opinions of those nurses who chose to complete the survey.

Another limitation of our study is its inability to identify whether the implementation of the addition of the “limitations of care” checkbox increased the proportion of patients whose limitations were amended during that admission than otherwise would have happened. Contextual elements that interacted with the intervention potentially included the frequent addition of new staff members (both nursing and physician). They may have varied experience and comfort with the end-of-life care, discussion, and documentation. Additionally, we know that disease patterns in pediatric critical care vary significantly over the year (eg, respiratory illnesses more common in the winter season, with trauma more common in the summer). Specific subsets of patients with limitations of life-sustaining therapies may have been more or less likely to be admitted at various points through the study period. We did not assess for any associations between our outcome measures and these potential contextual elements. However, we have demonstrated consistent documentation of limitations of life-sustaining therapies across several personnel changes and through multiple seasons, suggesting that these did not have a significant effect on our outcomes.

Factors that may have limited internal validity included the use of a convenience sample for the study and the lack of ability to corroborate the paper documentation with the electronic (gold standard) limitation of intervention orders. Although we were unable to modify the EMR related to limitations of life-sustaining therapies in this particular study, the data we have collected inform the work of another working group. This other group will continue to advocate for the more efficient integration of this information into the EMR. Future studies should investigate whether patients at higher risk of mortality (eg, technology-dependent or underlying oncological process or bone marrow transplant) are more likely to be impacted by this intervention than the patients at lower risk of mortality such as those with bronchiolitis. Additionally, although we used the term “limitations of care” on the Daily Goal Sheet, we would have selected another term, such as limitations of interventions, which we have used throughout the manuscript. This term is more reflective of the goals of limiting unnecessary interventions and prioritizing comfort without restricting the care we provide to patients.

## CONCLUDING SUMMARY

Limitations of life-sustaining therapies in the PICU are nuanced and involve multiple stakeholders. Nursing education and designation of limitations of life-sustaining therapies section on nursing Daily Goal Sheets can increase documentation of limitations in a PICU. Further studies should explore the sustainability of these interventions, expansion of this project to other institutions, and the impact on patient-centered outcomes, including prompting of more timely discussions between providers and families.

## DISCLOSURE

The authors have no financial interest to declare in relation to the content of this article.

## ACKNOWLEDGMENTS

Assistance with the study: Pediatric Intensive Care Unit Nurse champions: Laura Green, RN, BSN, CCRN; Mary Nelson, BSN, RN, CCRN; and Ashley Klass, RN, CCRN, CPN. This project was completed as part of the Fellow Core Curriculum Quality Improvement module, led by Drs. Christiane Corriveau and Padmaja Pavuluri.

## Supplementary Material


