# Supplementary material for: A Quality Improvement Project to Improve Documentation and Awareness of Limitations of Life-Sustaining Therapies
Source: Pediatr Qual Saf. 2020 May 28;5(3):e304. doi: 10.1097/pq9.0000000000000304 (PMC7297404; doi:10.1097/pq9.0000000000000304)
Supplement: Supplementary file 1 [file pqs-5-e304-s001.pdf]

Pre-Intervention Survey

Please complete the survey below.

Thank you!

This survey is designed to gauge our current practice of code status documentation and understanding in the PICU as part of a quality improvement project for the critical care fellows.

This is an anonymous survey. Results of the survey will be presented in aggregate data, with complete protection of individual anonymity.

Completion of this survey is entirely voluntary.

1) **Please indicate how much you agree with the following statement. As a bedside provider, I fully understand my patients' limitations of care (i.e. code status)**
   * must provide value

2) **Please indicate how much you agree with the following statement. The healthcare team always acts in accordance with a family's wishes when a patient has limitations of care in place.**

3) **Do you use visual signage or visual cues to remind you of a patient's limitations of care?**

4) **Would creation of a universal visual tool to identify patients with limitations in code status be helpful (i.e. placing a DNR sign in a patients room or creating a sign similar to the butterfly)?**

5) **In your opinion does "do not resuscitate" means the same thing for every patient?**

6) **Please share how code status is relayed in daily sign out in your discipline?**

   Expand

7) **When taking care of a patient with limitations of care, what things would be helpful for you to**

know?

Expand

8) **Please share any additional comments you have regarding communicating, understanding and documenting limitations of care.**

Expand

Submit

Powered by REDCap
