# Supplementary figures and images for: A Quality Improvement Project to Improve Documentation and Awareness of Limitations of Life-Sustaining Therapies
Source: Pediatr Qual Saf. 2020 May 28;5(3):e304. doi: 10.1097/pq9.0000000000000304 (PMC7297404; doi:10.1097/pq9.0000000000000304)

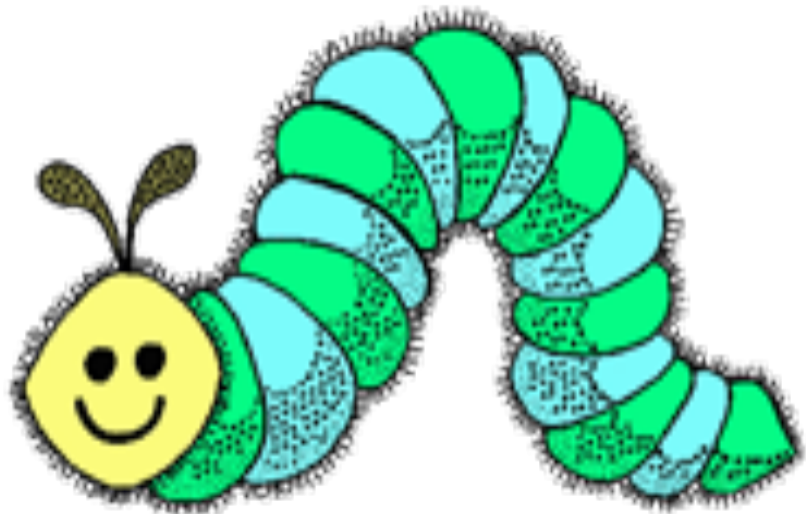

**Visual Cue for Patients with Care Limitations**

Supplement: Supplementary file 2 [file pqs-5-e304-s002.pdf]
